# Brassinosteroids Regulate Plant Growth through Distinct Signaling Pathways in Selaginella and Arabidopsis

**DOI:** 10.1371/journal.pone.0081938

**Published:** 2013-12-13

**Authors:** Jinyeong Cheon, Shozo Fujioka, Brian P. Dilkes, Sunghwa Choe

**Affiliations:** 1 School of Biological Sciences, College of Natural Sciences, Seoul National University, Seoul, Korea; 2 RIKEN Advanced Science Institute, Wako-shi, Saitama, Japan; 3 Department of Horticulture and Landscape Architecture, Purdue University, West Lafayette, Indiana, United States of America; 4 Convergence Research Center for Functional Plant Products, Advanced Institutes of Convergence Technology, Suwon, Gyeonggi, Korea; 5 Plant Genomics and Breeding Institute, Seoul National University, Seoul, Korea; University of Massachusetts Amherst, United States of America

## Abstract

Brassinosteroids (BRs) are growth-promoting steroid hormones that regulate diverse physiological processes in plants. Most BR biosynthetic enzymes belong to the cytochrome P450 (CYP) family. The gene encoding the ultimate step of BR biosynthesis in Arabidopsis likely evolved by gene duplication followed by functional specialization in a dicotyledonous plant-specific manner. To gain insight into the evolution of BRs, we performed a genomic reconstitution of Arabidopsis BR biosynthetic genes in an ancestral vascular plant, the lycophyte *Selaginella moellendorffii*. Selaginella contains four members of the CYP90 family that cluster together in the CYP85 clan. Similar to known BR biosynthetic genes, the Selaginella CYP90s exhibit eight or ten exons and Selaginella produces a putative BR biosynthetic intermediate. Therefore, we hypothesized that Selaginella CYP90 genes encode BR biosynthetic enzymes. In contrast to typical CYPs in Arabidopsis, Selaginella CYP90E2 and CYP90F1 do not possess amino-terminal signal peptides, suggesting that they do not localize to the endoplasmic reticulum. In addition, one of the three putative CYP reductases (CPRs) that is required for CYP enzyme function co-localized with CYP90E2 and CYP90F1. Treatments with a BR biosynthetic inhibitor, propiconazole, and *epi*-brassinolide resulted in greatly retarded and increased growth, respectively. This suggests that BRs promote growth in Selaginella, as they do in Arabidopsis. However, BR signaling occurs through different pathways than in Arabidopsis. A sequence homologous to the Arabidopsis BR receptor BRI1 was absent in Selaginella, but downstream components, including BIN2, BSU1, and BZR1, were present. Thus, the mechanism that initiates BR signaling in Selaginella seems to differ from that in Arabidopsis. Our findings suggest that the basic physiological roles of BRs as growth-promoting hormones are conserved in both lycophytes and Arabidopsis; however, different BR molecules and BRI1-based membrane receptor complexes evolved in these plants.

## Introduction

The characterization of Arabidopsis *dwarf* (*dwf*) mutants revealed that brassinosteroids (BRs) are essential for normal growth and development throughout the life cycle of the plant [1], [2]. Arabidopsis *dwf5* [3], *dwf7* [4], and *dwf1* [5], in an order of action in the biosynthetic pathways, are defective in the enzymatic steps that lead to the synthesis of campesterol, which serves as a precursor of BRs [2]. Campesterol is further modified by the BR biosynthetic enzymes defined by *de-etiolated2* [6], *dwf4* [7]–[9], *constitutive photomorphogenesis and dwarfism* [10], [11], and *BR6ox* [1], [12], [13]. Leaving a few steps including steroid-2-hydroxylase to be cloned, BR biosynthetic enzymes in the pathways have been mapped to a gene in Arabidopsis [2], [9].

Most of the BR biosynthetic enzymes belong to the cytochrome P450 (CYP) family [2]. CYP enzymes primarily act as monooxygenases to hydroxylate their substrates [14]. Plant CYPs form two primary clades, named A-type and non-A-type CYPs [15]. A-type CYPs mediate plant-specific metabolism, e.g., the synthesis of secondary metabolites such as lignin, whereas non-A-type CYPs participate in metabolic processes common to eukaryotes. CYP51, a sterol 14α-demethylase, is a representative ancestor of plant non-A-type CYPs [14]–[16]. CYP proteins are classified further into subgroups according to their sequence similarity: identity greater than 40% are categorized into the same numerical group and those that more than 55% into alphabetical subgroups [14], [17]. Arabidopsis BR biosynthetic CYPs belong to the CYP85 clan, which includes CYP90A1 (*cpd*), CYP90B1 (*dwf4*), CYP90C1 (*rotundifolia3*), CYP90D1, CYP85A1 (*BR6ox1*), and CYP85A2 (*BR6ox2*). In *Selaginella moellendorffii*, at least four genes cluster in the CYP85 clan, including CYP90E1, CYP90E2, CYP90E3, and CYP90F1 [14], [15], suggesting that BRs are biosynthesized using these putative BR biosynthetic enzymes.

BR signals are perceived by a plasma membrane-localized receptor kinase, BRASSINOSTEROID-INSENSITIVE1 (BRI1) [18]–[20]. BR binding activates the BRI1 kinase domain, switching its binding partner from BRI1 KINASE INHIBITOR 1 (BKI1) [21] to BRI1-ASSOCIATED-KINASE1 (BAK1) [22], [23]. The BRI1-BAK1 co-receptor then relays the phosphorylation signal to BR-SIGNALING KINASES (BSKs) [24] and CONSTITUTIVE DIFFERENTIAL GROWTH1 (CDG1) [25]. Upon receiving the BR signal, a type I protein phosphatase named BRI1 SUPPRESSOR 1 (BSU1) [2], [26] represses the function of BRASSINOSTEROID INSENSITIVE2 (BIN2), a GSK3β-like protein kinase that acts as an inhibitor in BR signaling pathways [27], [28]. BES1 and BZR1 are the substrates of BIN2; in the non-phosphorylated state, these transcription factors regulate the transcription of their target genes [29]. BR signaling results in the dephosphorylation of phosphorylated BZR1 by a phosphatase named PROTEIN PHOSPHATASE 2A (PP2A) [29], [30].

Whereas the BR biosynthetic and signaling pathways have been thoroughly characterized in Arabidopsis, our knowledge of these pathways is limited in other species. To gain insight into the evolution of BR pathways, were constituted Arabidopsis BR pathways in an ancestral vascular plant, the lycophyte *Selaginella moellendorffii*. Lycophytes constitute a monophyletic sister clade to the euphyllophytes, which includes ferns and seed plants. Paleobotanical evidence suggests that the two vascular plant lineages separated approximately ∼400 million years ago [31]. Most prominently, vascular patterning in the leaf-like structure (microphyll) of Selaginella is different from that in Arabidopsis, with Selaginella lacking leaf gaps. Furthermore, gibberellins are not the major growth-promoting hormone in Selaginella [32]. Because BRs are known to be important regulators of vascular patterning and growth stimulation in Arabidopsis, identifying the counterpart underlying genes in Selaginella by comparative genomics analysis would provide evolutionary insight into the roles of BR hormones.

## Results and Discussion

### Phylogenetic Analysis of Putative BR Biosynthetic Genes

In Arabidopsis, BRs are biosynthesized through the action of CYP proteins belonging to the CYP85 clan [33]. To establish how many of the BR biosynthetic enzymes are similar in Arabidopsis and Selaginella, we searched the Phytozome database for sequences in the Selaginella genome with similarity to BR biosynthetic CYPs in Arabidopsis. We identified four sequences showing significant similarity with Arabidopsis CYP85A1. Consistent with previous findings [34], all four of these sequences, which were previously named CYP90E1, CYP90E2, CYP90E3, and CYP90F1, clustered with CYP85A1.

According to the numbering convention of CYP sequences, the group number often reflects the catalytic function; for instance, CYP90s catalyze brassinosteroid biosynthetic reactions [16]. Arabidopsis CYP amino acid sequences ranged in identity from 32% to 83% with each other. A comparison of Arabidopsis and Selaginella CYP sequences revealed levels of identity as low as 31% between AtCYP85A1 and SmCYP90E1, and as high as 43% between AtCYP90A1 and SmCYP90F1 (Table 1).

**Table 1:** Percent similarity between amino acid sequences of CYPs.

	*Arabidopsis thaliana*							*Selaginella moellendorffii*			
		CYP85A2(BR6OX2)	CYP85A1(BR6OX1)	CYP90B1(DWF4)	CYP90A1(CPD)	CYP90C1(ROT3)	CYP90D1	CYP90E1	CYP90E2	CYP90E3	CYP90F1
***Arabidopsis*** ***thaliana***	**CYP85A2** **(BR6OX2)**	100									
	**CYP85A1** **(BR6OX1)**	83	100								
	**CYP90B1** **(DWF4)**	33	32	100							
	**CYP90A1** **(CPD)**	37	35	42	100						
	**CYP90C1** **(ROT3)**	33	33	37	42	100					
	**CYP90D1**	35	35	36	41	52	100				
***Selaginella*** ***moellendorffii***	**CYP90E1**	32	31	34	40	38	37	100			
	**CYP90E2**	34	32	33	38	39	37	86	100		
	**CYP90E3**	33	32	34	38	37	38	77	81	100	
	**CYP90F1**	37	36	38	43	39	35	35	37	37	100

www.ncbi.nlm.nih.gov/blast/bl2seq/wblast2.cgi). Percent identity between Arabidopsis CYP90s ranged from 32% (between AtCYP90B1 and AtCYP85A1) to 83% (between AtCYP85A1 and AtCYP85A2). A comparison of CYP sequences in Arabidopsis and Selaginella revealed identity values ranging from 31% (between SmCYP90E1 and AtCYP85A1) to 43% (between SmCYP90F1 and AtCYP90A1). SmCYP90E consists of three members that share 77∼86% identity, whereas SmCYP90F has one member, which has 35∼37% identity with the SmCYP90Es. Sequences of known BR biosynthetic enzymes from Arabidopsis were pair-wise compared with select sequences from Selaginella. Percent identity values were calculated using the 2 BLAST program (

**Figure 1:**
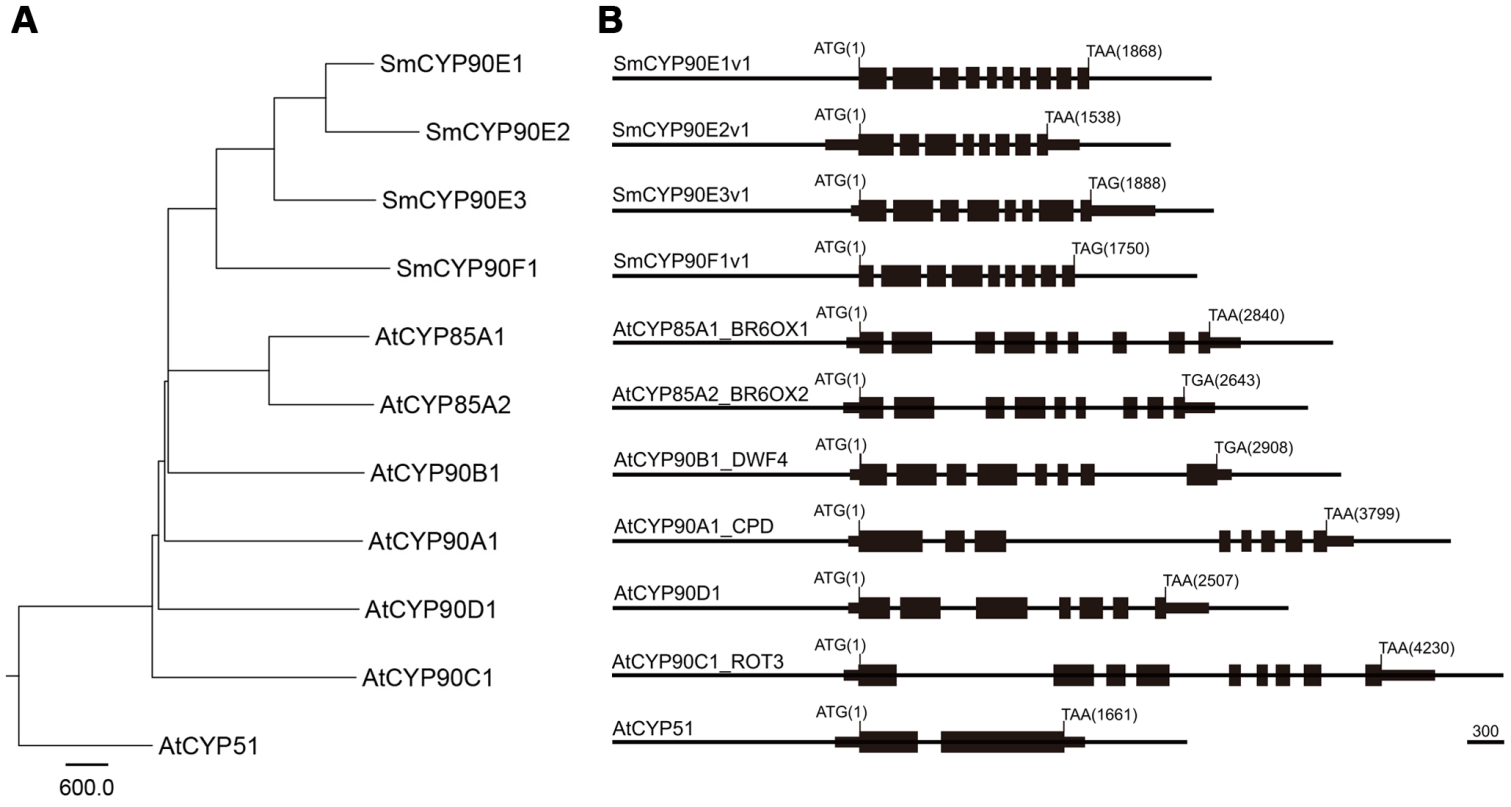
Phylogenetic tree and schematic representation of select CYP sequences. (A) Genomic DNA sequences were aligned using ClustalW, and the phylogenetic tree was generated using the Neighbor-joining method. The unit bar represents relative evolutionary distance. The Arabidopsis CYP51 sequence was used as an outgroup. (B) Schematic illustration of CYP genes. Filled boxes represent exons, and thinner boxes at the 5′ of 3′ ends indicate untranslated sequences. The base ‘A’ at the translation start codon was set to 1. All of the compared CYPs, except AtCYP51, consist of 7∼10 exons, characterizing BR biosynthetic genes belonging to the CYP85 clan.

To better understand the phylogenetic relationship between the CYP sequences, we generated a phylogenetic tree based on the genomic DNA sequences of the CYP genes (Figure 1A). We found that SmCYP90F1 is ancestral to the SmCYP90Es, and that SmCYP90E1 and SmCYP90E2 were duplicated relatively recently (Figure 1A). Selaginella CYPs clustered primarily with CYP90C1 and CYP90D1 of Arabidopsis, which catalyze the C-23 hydroxylation steps in the BR biosynthetic pathways [35], [36].

Previously, it was found that genes belonging to the CYP85 clan exhibit a scattered pattern of exons and introns [7]. For instance, AtCYP51A1 has only two exons separated by one intron [37], whereas all of the CYPs of the CYP85 clan possess seven to ten exons (Figure 1B). In contrast to the dispersed positioning of a relatively large number of exons in Arabidopsis CYPs, those of Selaginella had shorter introns, such that the length of genomic DNA spanning all of the exons and introns of Selaginella was more compact than that of Arabidopsis. In addition, we observed that the CYP genes often exhibited subtle differences in exon patterning; the seventh and eighth exons in the 5′ end of SmCYP90F1 were combined into a single exon in SmCYP90E3 (Figure 1B). The degree of sequence identity and similarity in the organization of the exons and introns suggests that the four CYP90s in Selaginella might be involved in BR metabolism. Future molecular complementation tests showing that Selaginella CYP90 sequences rescue a specific Arabidopsis BR dwarf mutant may pinpoint their function in BR biosynthesis.

### Subcellular Localization of CYP90E2 and CYP90F1

**Figure 2:**
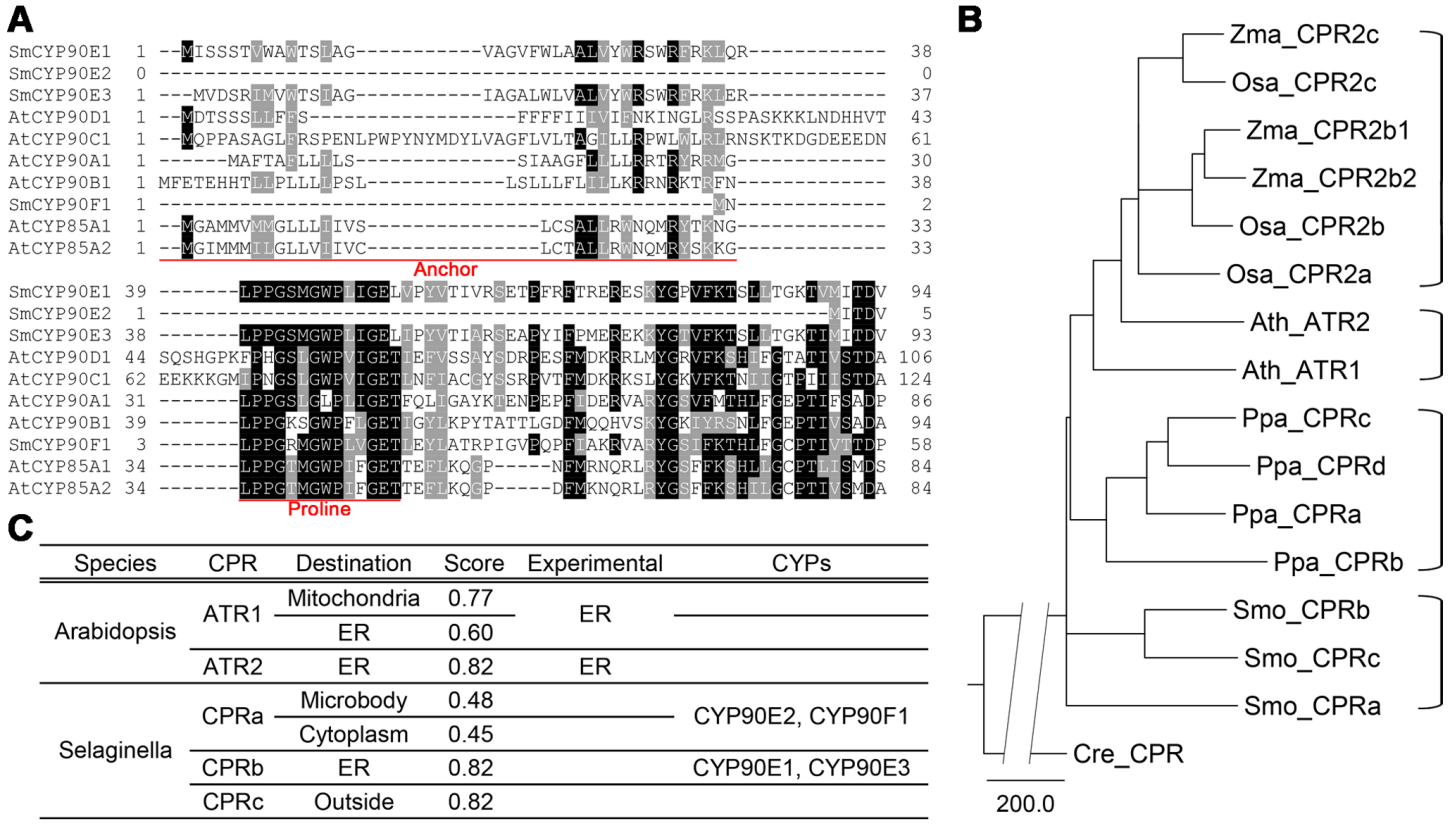
Predicted subcellular localization of CYPs and their putative reductases. (A) Multiple sequence alignment of CYP amino acid sequences from Selaginella and Arabidopsis. Sequences corresponding to functional domains identified in AtCYP90B1 are underlined. Box shading was carried out using BOXSHADE 3.21. Dashes represent gaps. Letters in black and gray backgrounds indicate 100% and 50% conservation among the sequences, respectively. The N-terminal regions up to the first 124 amino acids are shown. (B) Prediction of the subcellular localization of the NADPH-cytochrome P450 reductases (CPRs). The PSORT program was used to predict and score the calculation. (C) Phylogenetic tree of CPRs from Arabidopsis, maize, rice, Physcomitrella, and Selaginella. CPRs from the same species or from *Zea mays* (Zm) and *Oryza sativa* (Os) combined are bracketed on the right. The *Chlamydomonas reinhardtii* CPR sequence was used as an outgroup. The broken line for the outgroup indicates a genetic distance of 400. Scale bar = genetic distance of 200.

A multiple sequence alignment of the CYP sequences revealed that CYP90E2 and CYP90F1 lack signal peptides that are required for targeting to the endoplasmic reticulum (ER) (Figure 2A). Thus, we postulated that these two proteins might function elsewhere in the cell. To test this idea, we predicted the subcellular localizations of these proteins using the PSORT program. CYP90E1 and CYP90E3 were found to localize to the ER with a score of above 0.8, where 1 indicates full certainty; however, CYP90E2 and CYP90F1 had a greater probability of localizing to the microbody (peroxisome) or cytoplasm than to the ER (Figure 2B). Some ER-targeted proteins are known to lack typical signal sequences [38]. Since cytochrome P450reductases (CPRs) catalyze the transfer of electrons from NAD(P)H to CYPs and therefore co-localize with the CYPs [39], we would expect CPRs to co-localize with CYP90E2 and CYP90F1 at positions other than the ER.

Based on amino acid sequence similarity, we found that the Selaginella genome contains three putative *CPR* genes (Figure 2C), whereas Arabidopsis contains two and *Physcomitrella patens* four (Figure 2C). Interestingly, the PSORT program predicted that each of the three Selaginella CPRs (Smo_CPRs) have different cellular localizations; type A CPR (Smo_CPRa) is predicted to localize to the cytoplasm or microbody with similar certainty, Smo_CPRb to the ER, and Smo_CPRc to the extracellular matrix (outside) (Figure 2B). The finding that both the CYPs that lack signal sequences (i.e., CYP90E2 and CYP90F1) and their putative reductases are predicted to localize to positions other than the ER suggests that these CYPs function in subcellular positions other than the ER membrane.

### Brassinolide Stimulates Growth, whereas Propiconazole Suppresses Growth

**Figure 3:**
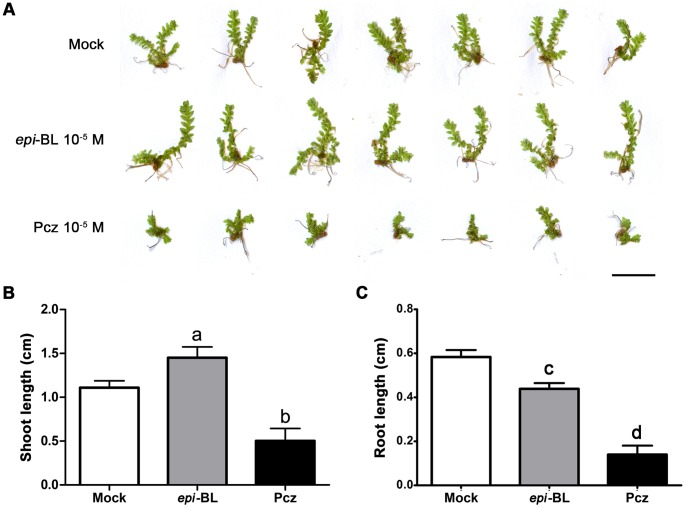
Response of Selaginella to *epi-*brassinolide (*epi-*BL) and propiconazole (Pcz). (A) Morphologies after treatment with mock, 10^−5^M *epi-*BL, and 10^−5^M Pcz for two weeks. Scale bar = 0.5 cm. (B) Average shoot lengths after treatment. Letters above each bar indicate significant differences compared with the mock treatment. Statistical significance was determined using one-way ANOVA (P<0.01, n = 7). Error bars are standard deviations. (C) Average root lengths after treatment. Letters above each bar indicate significant differences compared with the mock treatment. Statistical significance was determined using student t-test (P<0.01, n≥3). Error bars are standard deviations.

To understand how BRs affect Selaginella growth, we measured the growth responses of Selaginella after treatments with *epi*-brassinolide (*epi-*BL) and the BR biosynthetic inhibitor, propiconazole (Pcz) [40]. Compared with the mock control, the lengths of the shoots treated with 10^−5^ M *epi-*BL were longer after two weeks, whereas 10^−5^ M Pcz-treated seedlings were shorter (Figure 3A). Interestingly, the lengths of the two branches of mock-treated seedlings were similar, whereas *epi-*BL treatment tended to elongate one branch (Figure 3A). The elongation of the *epi-*BL-treated shoots was statistically significant (Figure 3B). Relative to the mock control, the shoots of plants treated with 10^−5^ M *epi-*BL were approximately 50% longer than those of the mock-treated control. However, the shoots of seedlings treated with10^−5^ M Pcz were approximately half as long as those of the control (Figure 3B). We measured the lengths of the roots, too. Different from the shoots, *epi-*BL-treated roots were significantly shorter than those of mock-treated plants. The Pcz-treated roots were also shorter than those of mock treatment. Inhibition of root growth by higher concentration of *epi-*BL is similar as what we observe in higher plants like Arabidopsis, suggesting that the physiological responses are conserved both in Selaginella and Arabidopsis. Considering that gibberellins do not induce significant elongation of Selaginella shoots [32], we hypothesize that BRs play a major role in Selaginella shoot growth.

### Analysis of Endogenous BRs

Based on our identification of putative Selaginella BR biosynthetic genes, it is likely that BRs are biosynthesized in Selaginella. We therefore searched for BRs in the aerial tissues of Selaginella; however, the levels of known BR biosynthetic intermediates were below detectable limits. Most BR intermediates found in Arabidopsis and rice were not detectable or only present at very low levels. However, there is still a possibility that uncommon BRs might be produced in Selaginella at greater levels than ordinary BRs. If this is the case, the chemical structures of the putative BRs will need to be determined before they can be quantified precisely.

### Similarity-based Identification of BR Signaling Genes

Once BRs are synthesized endogenously and act as signaling molecules, specific receptors perceive and transmit the signals. To determine if the signaling components of Arabidopsis also exist in Selaginella, we searched the Selaginella database using Arabidopsis BR signaling genes as probes. To identify homologs, we considered the degree of sequence identity, the phylogenetic tree, the probability determined in the BLAST analysis, and the domain structures of the protein sequences. Table 2 summarizes the findings of our BLAST search.

**Table 2:** Identification of BR signaling components by comparative sequence analysis.

Signaling component	Arabidopsis locus	Selaginella locus	Identity (%)	Probability (e value)
BRI1 (4)	At4G39400	99902	37.00	5.00E-166
BAK1 (5)	At4G33430	85471	73.70	0
		167872	58.70	3.50E-172
		268032	82.40	1.50E-171
		85818	55.80	5.50E-164
BKI1	At5G42750	–	–	–
BSK1 (3)	At4G35230	103506	72.80	0
CDG1	At3G26940	13751	61.60	7.00E-126
		99936	61.50	1.60E-124
		79963	53.10	2.50E-98
		151233	51.60	2.40E-93
		109804	50.50	7.10E-93
BIN2 (3)	At4G18710	159063	79.50	0
		132906	78.30	0
BSU1	At1G03445	438528	45.00	7.10E-180
BZR1	At1G75080	437216	77.80	2.6E-22
BES1	At1G19350	437216	79.20	8.50E-23
PP2A B’β	At3G09880	267276	74.10	1.00E-178
		63731	71.30	2.30E-168
		99313	70.40	6.20E-164
		149261	64.90	3.20E-150

’β (SERINE/THREONINE PROTEIN PHOSPHATASE 2A B’β). Percent identity values were obtained after performing a BLASTX search of the Phytozome database. The number of Arabidopsis gene copies is given in parentheses after each locus name. Abbreviations: BRI1 (BRASSINOSTEROID INSENSITIVE1), BAK1 (BRI1-ASSOCIATED RECEPTOR KINASE1), BKI1 (BRI1 KINASE INHIBITOR1), BSK1 (BR-SIGNALING KINASE1), CDG1 (CONSTITUTIVE DIFFERENTIAL GROWTH1), BIN2 (BRASSINOSTEROID-INSENSITIVE 2), BSU1 (BRI1 SUPPRESSOR1), BZR1 (BRASSINAZOLE-RESISTANT1), BES1 (BRI1-EMS-SUPPRESSOR1), BSK1 (BR-SIGNALING KINASE1), and PP2A B

**Figure 4:**
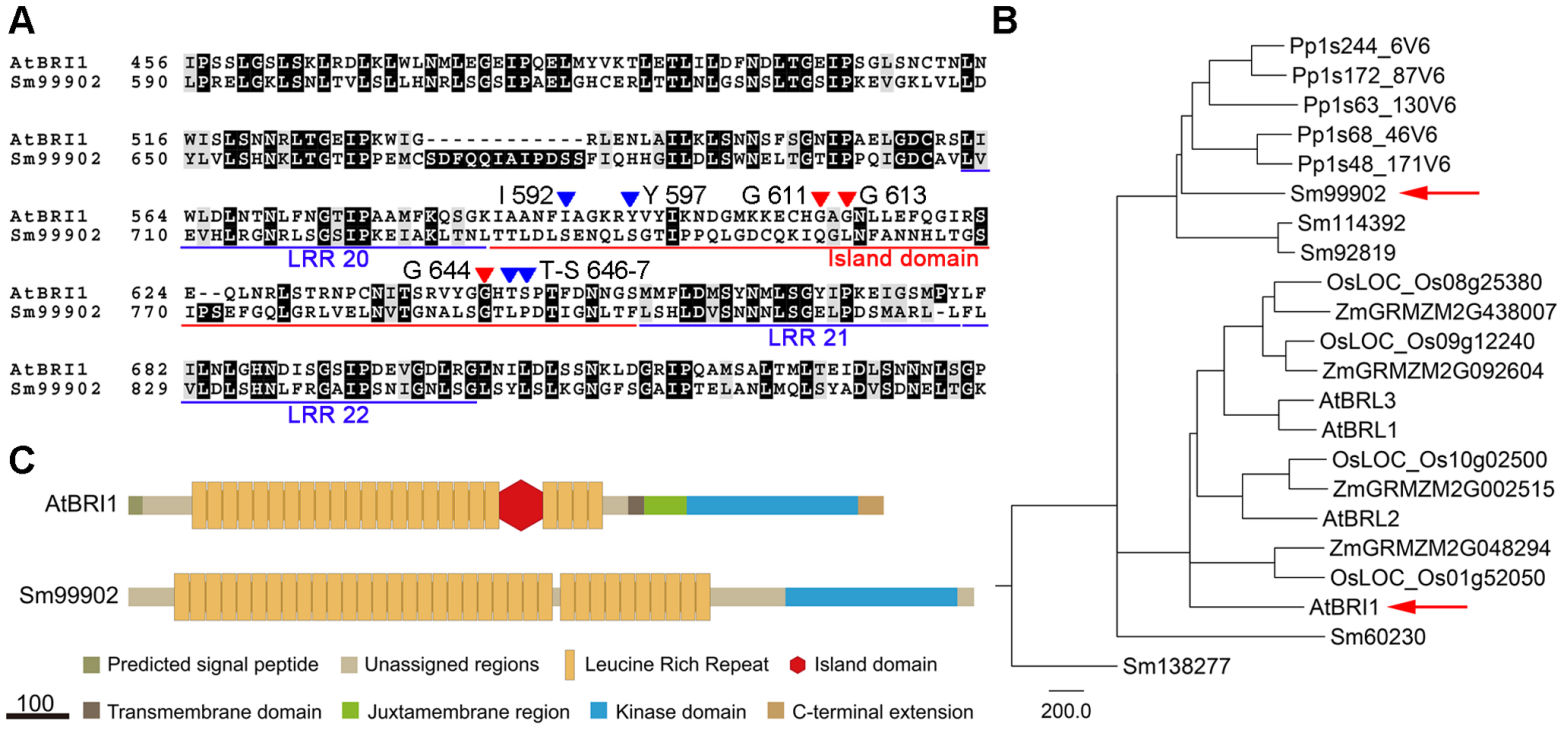
Comparison of Arabidopsis BRI1 (AtBRI1) with its closest homolog from Selaginella. (A) Multiple sequence alignment between AtBRI1 and the Selaginella sequence Sm99902 was performed using ClustalW. Underlining delimits the domains. Red triangles indicate residues identified as being functionally important by mutation studies [41] and blue triangles are key residues that participate in BL binding [42]. (B) Schematic representation of the domains identified in AtBRI1. A key for the colors and shapes used is provided below the diagram. Scale bar = 100 bp. (C) Phylogenetic tree of AtBRI1 and closely related sequences from maize, rice, Physcomitrella, and Selaginella. Arrows indicate AtBRI1 and Sm99902. Scale bar = genetic distance of 200.

Our results suggest that it is unlikely that Selaginella has a functional homolog of Arabidopsis BRI1. The most similar protein in Selaginella, 99902, shared only 37% sequence identity with Arabidopsis BRI1 (Table 2). The amino acid residues identified as being important by mutation analysis of the BR-binding island domain were not conserved in Selaginella (Figure 4A) [41], [42], suggesting that the Selaginella protein does not contain the island domain (Figure 4B). Phylogenetic analysis also showed that the most similar protein forms a separate cluster from Arabidopsis BRI1 and BRI1-like proteins (Figure 4C). Therefore, it is not likely that 99902 has similar functions to Arabidopsis BRI1 and BRI1-like proteins.

**Figure 5:**
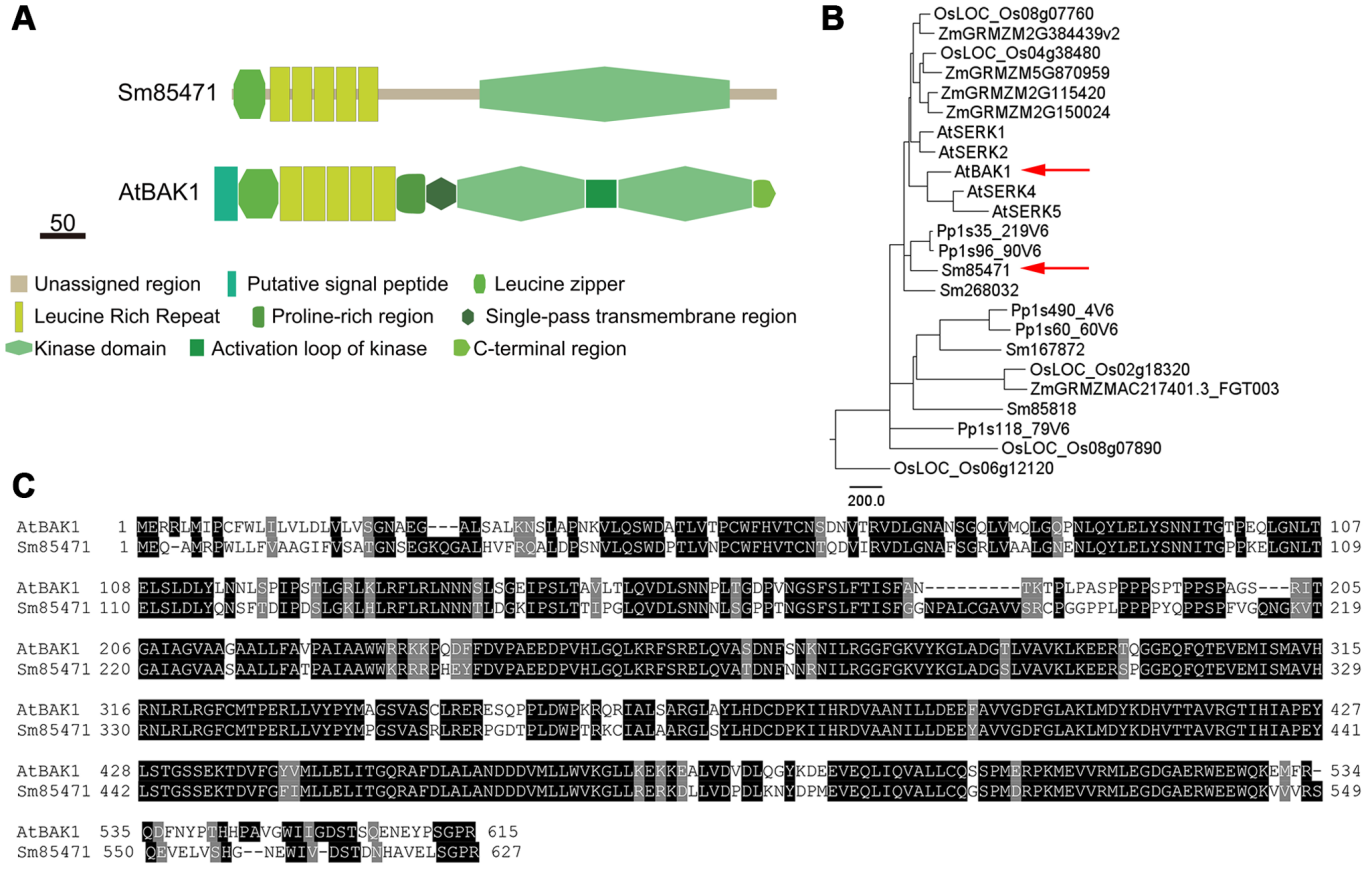
Comparison of Arabidopsis BAK1 (AtBAK1) with its homologs in Selaginella. (A) Functionally important domains, including the leucine-rich repeat and protein kinase domain, are conserved. (B) Phylogenetic analysis of BAK1-like protein sequences. Arrows indicate AtBAK1 and its close homolog Selaginella 85472 (Sm85471). (C) Multiple sequence alignment of AtBAK1 and Sm85471.

The Selaginella sequence most similar to Arabidopsis BAK1 was 85471, which shared 73% identity and a probability value of 0 (Table 2). A comparison of protein domains revealed that both BAK1 and 85471 have five leucine-rich repeats and a cytoplasmic kinase domain (Figure 5). These results suggest that 85471 is a homolog of Arabidopsis BAK1. However, in addition to its role as a BR co-receptor, BAK1 functions in the plant immune response [43]. Therefore, it would be interesting to conduct a functional analysis of 85471 to determine whether it also has multiple roles and to confirm its involvement in BR signaling.

It is likely that the Selaginella sequence 438528 is a homolog of Arabidopsis BSU1, because of its relatively high level of sequence identity (45%), low probability value (7.1E-180) (Table 2), conserved domain structure, and clustering pattern in the phylogenetic tree (Figure S1). Arabidopsis BSU1 functions as a protein phosphatase that uses BIN2 as a substrate to repress its negative role in signaling pathways. Furthermore, a homolog of BIN2 seems to exist in Selaginella. A Phytozome database search using Arabidopsis BIN2 as probe identified Selaginella sequence 159063, which had an e-value of 0. In addition, Selaginella counterparts of Arabidopsis BZR1, BSK1, and PP2A are present (Table 2).

In conclusion, BR biosynthetic pathways exist in Selaginella, based on the presence of putative BR biosynthetic genes (i.e., three CYP90Es and CYP90F). Interestingly, it appears that BR biosynthesis occurs in the cytoplasm in Selaginella, as neither CPRs nor two CYP proteins (i.e., CYP90E2 and CYP90F1) are targeted to the ER, which is the site of BR biosynthesis in Arabidopsis [38]. If BRs are indeed synthesized at positions other than the ER, the transport and secretion pathways of synthesized BRs is also expected to be different. One possibility is that the synthesized BRs are immediately used in the steroidogenic cells, instead of being transported out of the cells.

**Figure 6:**
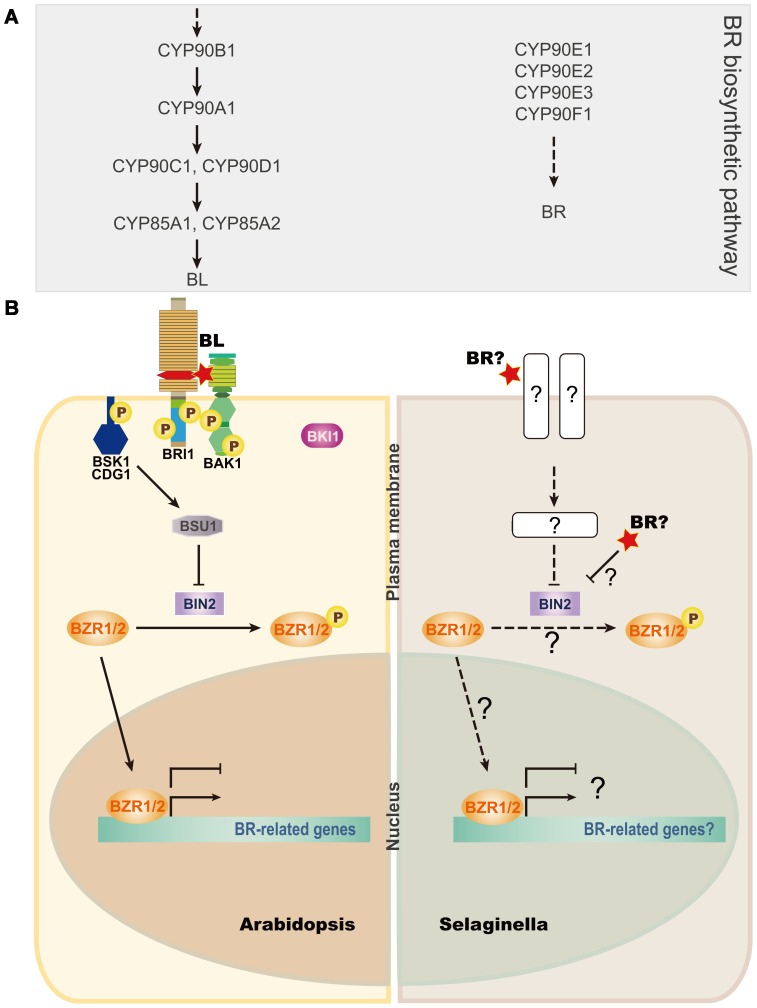
Parallel comparison of BR biosynthetic and signaling pathways. (A) Pathway showing the order in which Arabidopsis BR biosynthetic CYPs function. Selaginella CYP90s are shown to the right. (B) Model comparing Arabidopsis (left) with Selaginella (right) signaling pathways. Abbreviations are given beneath Table 2. P indicates phosphorylation. Solid lines indicate established signaling routes, whereas broken lines show possible routes. Flat-tipped and pointed arrows indicate inhibitory and positive actions, respectively. Question marks indicate steps that remain to be characterized.

Interestingly, we found that the typical membrane receptor complex for BRs in Arabidopsis, consisting of BRI1, BAK1, BKI, and BSK, is not present in Selaginella (Figure 6). However, the downstream signaling components, including BIN2, BSU1, and BZR1, seem to be present in Selaginella. Therefore, at least two possible scenarios could explain BR signaling in Selaginella. First, BRs may be perceived by a receptor complex that is possibly located in the plasma membrane, similar to that in Arabidopsis, and remains to be discovered. Second, BRs may be perceived directly by BIN2, by some unknown mechanism, and the signals may be transmitted to regulate gene expression through BR-specific transcription factors, including BZR1. In either case, it seems likely that BR biosynthetic and signaling pathways have evolved in Arabidopsis to include additional BR biosynthetic enzymes and a plasma membrane-localized receptor complex. In fact, it was shown that the ultimate step in the BR biosynthetic pathways in Arabidopsis is absent in monocot plants, including rice [44], suggesting that BR biosynthetic pathways evolved to meet the needs of individual plant species. Functional analyses of the putative BR genes in Selaginella identified here will provide insight into the evolution and roles of BRs in Selaginella and Arabidopsis.

## Methods

### Plant Growth and Morphometric Analysis

Aerial tissues of *Selaginella moellendorffii* grown in soil for two years in a greenhouse were harvested for analysis of endogenous BR levels. The tissues, including strobili, microphylls, ligules, spores, and bulbils, were extracted twice with 300 mL of methyl alcohol, and spiked with deuterium-labeled internal standards for quantification of BR biosynthetic intermediates. Purification and quantification of BRs were performed according to a previously described method [45].

For sterile growth, bulbils were sterilized for 12 min by shaking in a sterilization solution consisting of 50% Clorox and 0.1%Triton X-20, and then washed five times with sterile water. The bulbils were sprinkled on 0.65% agar-solidified media containing 0.5X Murashige and Skoog salts (pH 6.5). Plates containing bulbils were grown on the bench top at room temperature and illuminated with light at 7 µmol m^−2^ s^−1^. After two weeks of growth, germinated bulbils were transferred to media supplemented with 10^−5^ M propiconazole (Pcz) or *epi-*brassinolide (*epi-*BL). Bulbils were grown on the supplemented media for two more weeks and then subjected to morphometric analysis.

### Phylogenetic and Schematic Analysis

The amino acid sequences of the genes involved in the BR biosynthetic and response pathways were extracted from the Phytozome database (http://www.phytozome.net). Using the ClustalW function of the Jalview program in Phytozome, a multiple sequence alignment was performed and analyzed, and phylogenetic trees were generated using the Neighbor-joining method based on a BLOSOM62 matrix. To visualize the phylogenetic trees, the Figtree program was used [46].

To draw the exon map shown in Figure 1, the cDNA sequences were aligned with the genomic DNA sequences to delimit the borders of exons and introns. To estimate the subcellular localizations of the proteins, the protein sequences were submitted to the PSORT program (http://psort.hgc.jp/). The results are summarized in Figure 2B. To analyze functional similarities of the compared proteins, schematics of the proteins showing defined protein domains were generated [7], [47], [48].

## Supporting Information

Figure S1 **Comparison of Arabidopsis BSU1 (AtBSU1) and BES1 (AtBES1) with their close homologs in Selaginella.** (A) Schematic comparison of AtBSU1 and Selaginella 438528 (Sm438528). (B) Phylogenetic analysis of BSU1-like protein sequences. Arrows indicate AtBSU1 and its close homolog Sm438528. (C) Schematic comparison of AtBES1 and Selaginella 437216 (Sm437216). (D) Phylogenetic analysis of BES1-like protein sequences. Arrows indicate AtBES1 and its close homolog Sm437216. Scale bars represent 100 and 50 bp, respectively, in (A) and (C). Scale bar = genetic distance of 200.
(TIF)
