# Supplementary figures and images for: Brassinosteroids Regulate Plant Growth through Distinct Signaling Pathways in Selaginella and Arabidopsis
Source: PLoS One. 2013 Dec 13;8(12):e81938. doi: 10.1371/journal.pone.0081938 (PMC3862569; doi:10.1371/journal.pone.0081938)

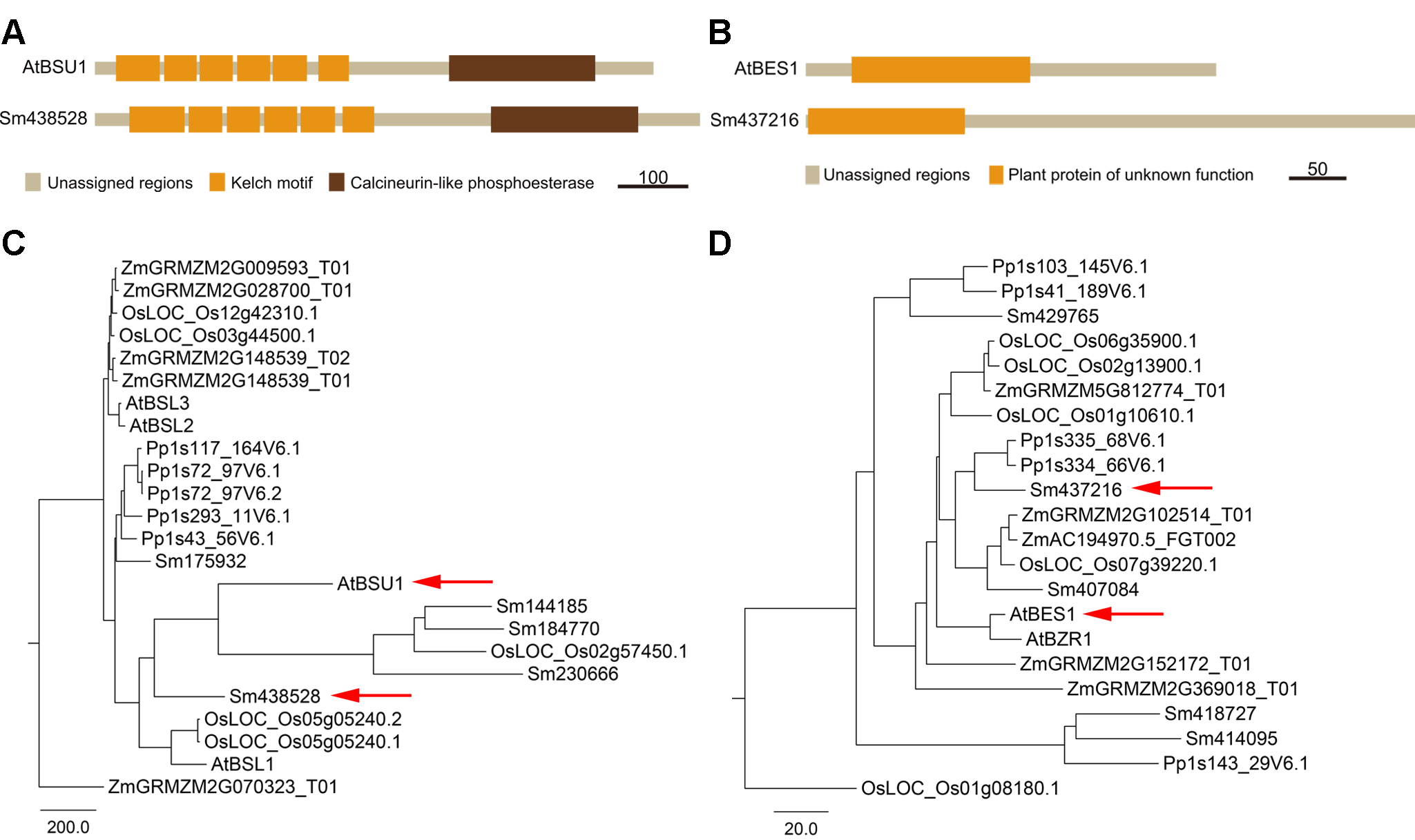

Supplement: Figure S1 — Comparison of Arabidopsis BSU1 (AtBSU1) and BES1 (AtBES1) with their close homologs in Selaginella. (A) Schematic comparison of AtBSU1 and Selaginella 438528 (Sm438528). (B) Phylogenetic analysis of BSU1-like protein sequences. Arrows indicate AtBSU1 and its close homolog Sm438528. (C) Schematic comparison of AtBES1 and Selaginella 437216 (Sm437216). (D) Phylogenetic analysis of BES1-like protein sequences. Arrows indicate AtBES1 and its close homolog Sm437216. Scale bars represent 100 and 50 bp, respectively, in (A) and (C). Scale bar = genetic distance of 200. (TIF) [file pone.0081938.s001.tif]
